# Symptom rates and profile clustering in tuberous sclerosis complex-associated neuropsychiatric disorders (TAND)

**DOI:** 10.1186/s11689-021-09408-8

**Published:** 2021-12-13

**Authors:** Samuel Alperin, Darcy A. Krueger, David N. Franz, Karen D. Agricola, Gabrielle Stires, Paul S. Horn, Jamie K. Capal

**Affiliations:** 1 Department of Neurology, Cincinnati Children’s Hospital Medical Center, Cincinnati, OH USA; 2 University of Cincinnati College of Medicine, Cincinnati, OH USA; 3 University of North Carolina at Chapel Hill, Chapel Hill, NC USA

**Keywords:** Tuberous sclerosis complex, TSC-associated neuropsychiatric disorders (TAND), Intellectual disability

## Abstract

**Background:**

Tuberous Sclerosis Complex (TSC) is associated with a range of neuropsychiatric difficulties, appropriately termed TSC-Associated Neuropsychiatric Disorders (TAND). The objectives of the study were to analyze the rates of TAND symptoms in a cohort of patients seen at the TSC Center of Excellence at Cincinnati Children’s Hospital and to identify clinically meaningful profiles based on TAND symptoms.

**Methods:**

Data from the TAND Checklist was obtained from participants seen at the TSC Center of Excellence at Cincinnati Children’s Hospital Medical Center from June 2015 to August 2018. Cluster and factor analyses for each TAND symptom were performed. Factor scores were then calculated for participants, and a K-means cluster analysis of these scores was used to empirically identify distinct overall TAND symptom profiles occurring in TSC.

**Results:**

A total of 1545 checklists was completed for 668 participants (37% adults and 63% children). Approximately 90% of participants reported at least one TAND symptom with an average of 12 symptoms (out of 29). Symptom rates ranged between 5 and 60%. The most common symptoms were neuropsychologic symptoms. A seven-cluster and seven-factor solution were found to be optimal. K-means cluster analysis resulted in a seven-profile solution, ranging from low to high symptom burden.

**Conclusion:**

This study is the first to identify natural phenotypic profiles of TAND symptoms. Study of specific TAND subpopulations with shared profiles may facilitate better understanding of the underlying biology of TAND and better assessment of more targeted treatments.

## Introduction

Tuberous-sclerosis complex (TSC) is an autosomal-dominant genetic disorder caused by pathogenic variants in *TSC1* and *TSC2*, affecting about 1 in 6000 live births and about 1 million people worldwide [1]. TSC affects all major organ systems with high variability in disease severity. TSC is also associated with a range of behavioral, intellectual and academic, psychiatric, neuropsychological, and psychosocial difficulties impacting approximately 90% of individuals with TSC and representing a significant burden [2, 3]. It has been shown that mental health is a significant part of the disease and that cost is a major barrier to improvement [4].

To improve identification of neuropsychiatric difficulties experienced by individuals with TSC, the Neuropsychiatric Panel at the 2012 TSC Consensus Conference developed the term TAND (TSC-associated Neuropsychiatric Disorders) [5]. TAND encompasses six “levels” of investigation: (1) behavioral level, (2) psychiatric level, (3) intellectual level, (4) academic level, (5) neuropsychological level, and (6) psychosocial level [3]. TAND symptoms are highly prevalent in patients with TSC overall, yet only 20% of patients receive appropriate assessment and treatment [3]. The TuberOus Sclerosis registry to increase disease Awareness study (TOSCA), a large international registry capturing the natural history of TSC, noted high rates of missing and unreported TAND data presumably from lack of assessment and treatment, sometimes greater than 60% for behavioral symptoms and intellectual levels [6].

TSC Consensus Conference recommendations include that individuals with TSC be assessed for TAND every year with the goal of lessening the identification gap between symptoms and recognition and identification by clinicians [3, 5]. With this consensus in mind, the TAND Checklist-L was developed as a screening tool to guide clinicians in discussing the different levels of TAND [3]. The checklist includes a total of 11 sections, with mostly yes/no questions and covers each level of investigation and was validated by *Leclezio* and colleagues in 2014 [7].

Leclezio and colleagues also attempted to identify natural grouping of symptoms or “clusters” with the goal of identifying groups of specific characteristics across levels of TAND and subsequently enabling healthcare providers to implement appropriate treatments focused on individual clusters/symptom groups. Initially, a feasibility study was completed (with 56 participants from South Africa and Australia) [8], followed by a larger replication study (with 85 participants from the TOSCA registry) which showed a six-cluster solution [9]. These were namely (1) scholastic, (2) neuropsychological, (3) mood/anxiety, (4) ASD-like, (5) challenging behaviors, and (6) hyperactive/impulsive [8, 9]. In a larger study with bootstrapping and internal consistency, a final seven-cluster solution was selected, including the addition of one for eating/sleeping that previously had been divided into the ASD-like cluster (eating) and mood/anxiety cluster (sleeping) rather than its own stand-alone cluster [10].

Using a cohort of participants seen at the TSC Center of Excellence at Cincinnati Children’s Hospital Medical Center (CCHMC), we aimed to analyze rates of TAND symptoms in TSC patients evaluated and treated at a large multidisciplinary TSC referral center in the USA. We then aimed to replicate the cluster and factor analyses to identify similar symptom clusters as found in prior studies by *Leclezio* et al. Finally, we aimed to extend this analysis and identify clinically meaningful symptom profiles based on TAND symptoms.

## Methods

### Participant recruitment and study design

Participants consisted of patients seen at the Tuberous Sclerosis Clinic Center of Excellence at CCHMC from June 2015 to August 2018. As part of the clinic, the TAND checklist was administered via tablet (iPad) during the visit and was completed by either the participant or a parent/caregiver at each clinic visit. Parents completed the checklist for participants who were minors (<18 years old) or had intellectual disability. TAND Checklist responses are immediately available within the patient’s electronic health record, and clinicians reviewed and discussed the responses with the family at the same visit. All participants with a TSC diagnosis and who completed the TAND checklist were eligible to be included in the study. Checklists that had >50% missing data were excluded from analysis. The CCHMC Institutional Review Board approved the protocol through which this research was performed. Informed consent was obtained from adult participants and from parents or guardians for participants under 18 years of age. Assent was provided by participants when able per institutional requirements.

### TAND checklist

The TAND Checklist [3] contains 12 sections: (1) basic developmental milestones; (2) current level of function; (3) behavioral concerns; (4) diagnosed psychiatric disorders; (5) intellectual ability; (6) academic skills; (7) neuropsychological skills; (8) psychosocial functioning; (9) parent, caregiver, or self-rating of impact of TAND; (10) prioritization list; (11) additional concerns; and (12) health-care professional rating of impact of TAND. We focused only on sections 1–10 of the TAND Checklist for the current analysis, as these are the only portions of the TAND Checklist that are captured directly from patient/caregiver input at CCHMC via tablet integration to clinical visits as described above.

### Data analysis

All analyses were performed with the R software package [11]. Section 3 (Behavior Challenges), section 4 (Psychiatric Disorders), section 6 (Academic Skills), and section 7 (Neuropsychological Skills) were included in analyses of rates (both at first available checklist and over the subsequent visits). However, only sections 3, 6, and 7 were included in the cluster and factor analyses, maintaining consistency with previous analyses by Leclezio et al. and De Vries et al*.* [9, 10]. Given that TAND Checklist variables were binary, correlation matrix for the 29 variables using the first available visit for each participant was computed with mean-squared contingency coefficient (missing values were omitted pairwise in correlation computations). Impact of TAND symptoms was assessed via Section 9, which asked “how much have these bothered, troubled, or distressed you/your child/family,” and ranged between 0 (“not at all”) and 10 “extremely.” Each participant (or guardian) received a separate “Seizure questionnaire.” This was not done using a standardized instrument, but was rather collected as part of their routine clinical care. However, to increase the consistency and content of seizure frequency documentation in clinical notes, a uniform clinical flowsheet was utilized within the electronic medical health record created at each clinical encounter. The flowsheet specifically prompts the patient or caregiver to respond to the questions, “I/my child on average has ____ (number) of seizures per _____ (day/week/month/year).” *P* values were considered significant if they were below 0.05 after false discovery rate (FDR) adjustment.

Different methods of cluster analyses were then performed. Initially, hierarchical clustering methods were used to suggest suitable number of clusters. Multiple methods were used, including complete linkage, average linkage, Ward’s method, and McQuitty’s method (via the hclust() R function). Other functions that were used included PAM (partitioning around medoids, an extension of k-means clustering), FANNY (fuzzy clustering, based on probability of cluster belonging), and DIANA (divisive analysis, clusters are formed by dividing larger ones) [12]. The best cluster size and method was chosen based off optimizing statistics like gap, within cluster sums, and elbow method.

Exploratory factor analysis was then completed using the function fa() from the R package *psych* [13]*.* Multiple methods with different factor extractions (total of five methods) and rotation methods (total of fourteen) were used, and combinations of each were applied, again maintaining consistency with previous analyses by Leclezio et al. and De Vries et al. [9, 10]. The Tucker index of factor congruence for each combination was used to find a factor solution most like our chosen best cluster analysis [14]. Using the Cronbach’s alpha, internal consistency was tested for each cluster and factor, with 0.70 considered good internal consistency.

Once a suitable factor analysis was obtained, factor scores were calculated for all participants. K-mean cluster analysis of the factor scores for each participant was then used to estimate empirical symptom profiles. Optimal number of clusters/profiles was defined as the number that maximized explained variance in factor scores without producing small splinter clusters.

## Results

A total of 1545 checklists were completed for 668 participants (250 (37%) adults and 418 (63%) children) within the 3-year time period (an additional 99 records were removed for > 50% missing data). Demographics for the cohort can be seen in Table 1. Approximately 37% of the participants were only seen one time. Of those seen for multiple visits, the average interval between appointments was 324 days, with 70.8% seen within one year of previous appointment (73% in children and 67% in adults). Of note, 65% of the participants did not self-report intellectual ability (as reported by themselves, parent, or caregiver of previous formal evaluation). Of those who did self-report, about 38.5% of the participants had normal intellectual ability, with another 40% with mild-moderate intellectual disability. Parental/guardian assessment of intellectual ability and official IQ testing was generally consistent (weighted-kappa *κ* = 0.72, 95% CI 0.68–0.77).

**Table 1:** Demographics

	Children (<18 years old) *n* = 418	Adults (≥ 18 years old ) *n* = 250	Total *n* = 668
**Age**			
<4 **years old** (total visits)	130 (252)	--	--
4–11 **years old** (total visits)	177 (417)	--	--
12–18 **years old** (total visits)	111 (287)	--	--
18–40 **years old** (total visits)	--	202 (504)	--
>40 **years old** (total visits)	--	48 (85)	--
**Total number of visits**	956	589	1545
**Sex**			
Male	228 (55%)	118 (47%)	346 (52%)
Female	190 (45%)	132 (53%)	322 (48%)
**Number of visits per patient**			
Average number (SD, range)	2.28 (±1.34, 1–7)	2.36 (±1.28, 1–8)	2.31 (±1.32, 1-8)
Only 1 visit (% of total)	161 (38.5%)	84 (33.6%)	245 (36.7%)
**Days between visits**			
Average (±SD, range)	316.9 (±316.9, 14–1120)	335.2 (±169.17, 42–1120)	324 (±164.67, 14-1120)
**Follow-up within 1 year**	394 out of 538 (73.4%)	227 out of 339 (67.0%)	621 of 877 (70.8%)
**Intellectual ability (self-report)**^a^ *(n = 234)*			
Normal (IQ ≥70)	45 (35.4%)	45 (42.1%)	90 (38.5%)
Mild-moderate (IQ 35–69)	57 (44.9%)	36 (33.6%)	93 (39.8%)
Severe profound (IQ < 34)	25 (19.7%)	26 (24.3%)	51 (21.8%)
Unknown	291 (69.6%)^b^	143 (57.2%) ^b^	434 (65.0%) ^b^

^a^Self-report of formal evaluation, per Section 5, question of TAND checklist

^b^Percent of total patients in each column

Six hundred two individuals (90%) reported at least one TAND symptom at the first visit. The average number of symptoms at the first visit was approximately twelve. Rate of each TAND symptom at the initial visit can be seen in Table 2. The most common symptom was neuropsychologic difficulty with attention (59.4%). Other commonly reported symptoms were scholastic, neuropsychological (namely difficulties with multitasking and other executive skills), and behavioral (namely difficulties with attention, mood swings, and anxiety), all of which were present in more than half the participants at their first visit. When divided by age, the most common symptoms for adults were anxiety, depression, mood swings, sleep, and memory problems (all statistically more common than in children). Most common symptoms for children were temper tantrums, overactivity, impulsivity, and scholastic difficulties (often statistically more common than in adults). There was not a significant increase in rate of symptoms over the subsequent visits, with biggest increase noted for sleep difficulties (+1.6%).

**Table 2:** Rate of symptoms by age (child versus adult) for first visit and lifetime

Section	Symptom	Total rate (%)	Child rate (%)	Adult rate (%)	Child versus adult ***χ***^**2**^ (***p value)***^a,b^	Lifetime frequency^c^ (%)
**Section 3**	Anxiety	52.5	43.3	**68.0**	38.27 (<0.0001) *	53.0
	Depressed mood	30.2	17.0	**52.4**	93.00 (<0.0001) *	31.0
	Extreme shyness	21.6	19.6	24.8	2.48 (0.11)	21.9
	Mood Swings	52.4	47.8	**60.0**	9.26 (0.002) *	52.7
	Aggressive outburst	42.1	40.9	44.0	0.61 (0.43)	43.3
	Temper tantrums	42.5	**48.6**	32.4	16.73 (<0.0001) *	32.7
	Self-injury	25.1	26.3	23.2	0.81 (0.37)	25.4
	Delayed language	47.2	**51.0**	40.8	6.48 (0.01) *	48.2
	Repeats words	35.6	35.6	35.6	0.00 (0.99)	36.4
	Poor eye contact	36.7	36.6	36.8	0.00 (0.96)	36.8
	Difficulty with peers	32.5	33.0	31.6	0.14 (0.71)	32.8
	Repetitive behaviors	41.2	41.1	41.2	0.00 (0.99)	41.5
	Rigid/inflexible	43.4	40.7	48.0	3.42 (0.06)	44.2
	Overactive	34.9	**40.2**	26.0	13.87 (0.0002) *	35.2
	Difficulties with attention	57.3	56.9	58.0	0.07 (0.79)	58.2
	Restlessness	46.1	48.1	42.8	1.76 (0.18)	46.7
	Impulsivity	44.3	**47.6**	38.8	4.92 (0.03) *	45.5
	Difficulty with eating	36.8	38.0	34.8	0.71 (0.40)	37.7
	Sleep difficulties	46.7	41.4	**55.6**	12.69 (0.0004) *	48.4
**Section 4**^d^	ASD	23.4	24.3	22.0	0.49 (0.48)	23.6
	ADHD	21.9	**24.8**	17.1	5.41 (0.02) *	22.4
	Anxiety Disorder	19.1	13.3	**29.0**	24.67 (<0.0001) *	19.7
	Depressive	13.8	5.3	**28.3**	68.16 (<0.0001) *	14.1
	OCD	13.1	10.1	**18.0**	8.48 (0.004) *	13.5
	Psychotic	4.9	2.2	**9.4**	17.52 (<0.0001) *	5.3
**Section 6**	Reading	41.1	**44.4**	35.4	4.97 (0.03) *	41.9
	Writing	38.8	**43.2**	31.2	9.03 (0.003) *	39.3
	Spelling	37.6	**41.0**	31.6	5.57 (0.02) *	38.0
	Mathematics	43.3	42.7	44.3	0.16 (0.69)	44.0
**Section 7**	Memory	36.6	31.0	**46.4**	15.41 (<0.0001) *	38.0
	Attention	59.4	57.9	62.0	1.07 (0.30)	60.3
	Multi-tasking	58.1	58.0	58.2	0.00 (0.96)	58.9
	Visuo-spatial tasks	38.8	41.0	35.0	2.24 (0.13)	39.7
	Executive skills	54.5	56.3	51.5	1.40 (0.24)	55.0
	Getting disoriented	33.0	31.6	35.4	1.00 (0.32)	33.8

^a^*represents *p* value that was still significant (<0.05) after FDR-adjustment. The higher prevalence between adult and child groups is bolded

^b^Chi-square analysis with 1 degree of freedom

^c^Frequency of “yes” throughout study period

^d^Section 4 was not included in the cluster or factor analyses

Symptom rate by IQ was also analyzed for the 234 participants for whom a formal IQ level was known (Table 3). Most symptoms at the behavioral, intellectual, academic, and neuropsychological levels were significantly less common in those without intellectual disability (IQ ≥ 70) compared to those with intellectual disability (IQ < 70). The exception to this relationship was the psychiatric level, where only the diagnosis of autism spectrum disorder (ASD) and intellectual disability (IQ < 70) remained significantly associated with one another.

**Table 3:** Prevalence of symptoms by self-reported IQ level

Section	Symptom	Normal (IQ ≥70) *n = 90 (%)*	Mild/moderate disability (IQ 35–69) *n = 93 (%)*	Severe/ profound disability (IQ <35) *n = 51 (%)*	Normal vs abnormal IQ ***χ***^**2**^ (***p value***)^a,b,c^
**Section 3**	Anxiety	67.8	74.2	58.8	0.02 (0.88)
	Depressed mood	44.4	40.9	25.5	1.90 (0.17)
	Extreme shyness	32.2	25.8	17.6	2.46 (0.12)
	Mood Swings	54.4	64.5	68.6	3.11 (0.08)
	Aggressive outburst	38.9	55.9	66.7	9.63 (0.002) *
	Temper tantrums	33.3	57.0	58.8	13.10 (0.0003) *
	Self-injury	14.4	25.8	58.8	14.41 (< 0.0001) *
	Delayed language	27.8	62.4	88.2	42.78 (< 0.0001) *
	Repeats words	28.9	63.4	56.9	23.02 (< 0.0001) *
	Poor eye contact	28.9	46.2	60.8	11.46 (0.0004) *
	Difficulty with peers	34.4	55.9	49.0	8.07 (0.0007) *
	Repetitive behaviors	34.4	57.0	76.5	19.26 (< 0.0001) *
	Rigid/inflexible	44.4	67.7	66.7	11.98 (0.0005) *
	Overactive	31.1	41.9	51.0	4.55 (0.03) *
	Difficulties with attention	58.9	78.5	86.3	13.94 (0.0002) *
	Restlessness	42.2	51.6	76.5	7.37 (0.007) *
	Impulsivity	40.0	71.0	64.7	18.76 (< 0.0001) *
	Difficulty with eating	27.8	39.8	72.5	12.65 (0.0004) *
	Sleep difficulties	56.7	63.4	56.9	0.45 (0.50)
**Section 4** ^d^	ASD	17.8	37.6	51.0	15.16 (< 0.0001) *
	ADHD	34.4	41.9	27.5	0.13 (0.71)
	Anxiety disorder	32.2	31.2	17.6	0.92 (0.34)
	Depressive	21.1	25.8	11.8	0.00 (0.96)
	OCD	16.7	26.9	25.5	2.99 (0.08)
	Psychotic	3.3	9.7	11.8	3.91 (0.05) *
**Section 6**	Reading	40.0	78.3	62.0	24.28 (< 0.0001) *
	Writing	34.4	75.0	62.0	29.01 (< 0.0001) *
	Spelling	30.0	70.7	64.0	32.49 (< 0.0001) *
	Mathematics	33.3	85.9	64.0	46.45 (< 0.0001) *
**Section 7**	Memory	37.8	52.7	68.0	9.13 (0.001) *
	Attention	60.0	86.8	82.0	18.63 (< 0.0001) *
	Multi-tasking	58.9	93.4	88.0	34.93 (< 0.0001) *
	Visuo-spatial tasks	25.6	58.2	80.0	35.87 (< 0.0001) *
	Executive skills	53.3	91.2	84.0	36.44 (< 0.0001) *
	Getting disoriented	20.0	49.5	72.0	31.46 (< 0.0001) *

^a^Abnormal is defined as anyone with IQ < 70 (includes mild, moderate, severe, and profound groups)

^b^Chi-square analysis with 1 degree of freedom

^c^*represents *p* value that stayed significant (< 0.05) after FDR-adjustment

^d^Section 4 was not included in the cluster or factor analyses

### Cluster analysis

**Fig. 1:**
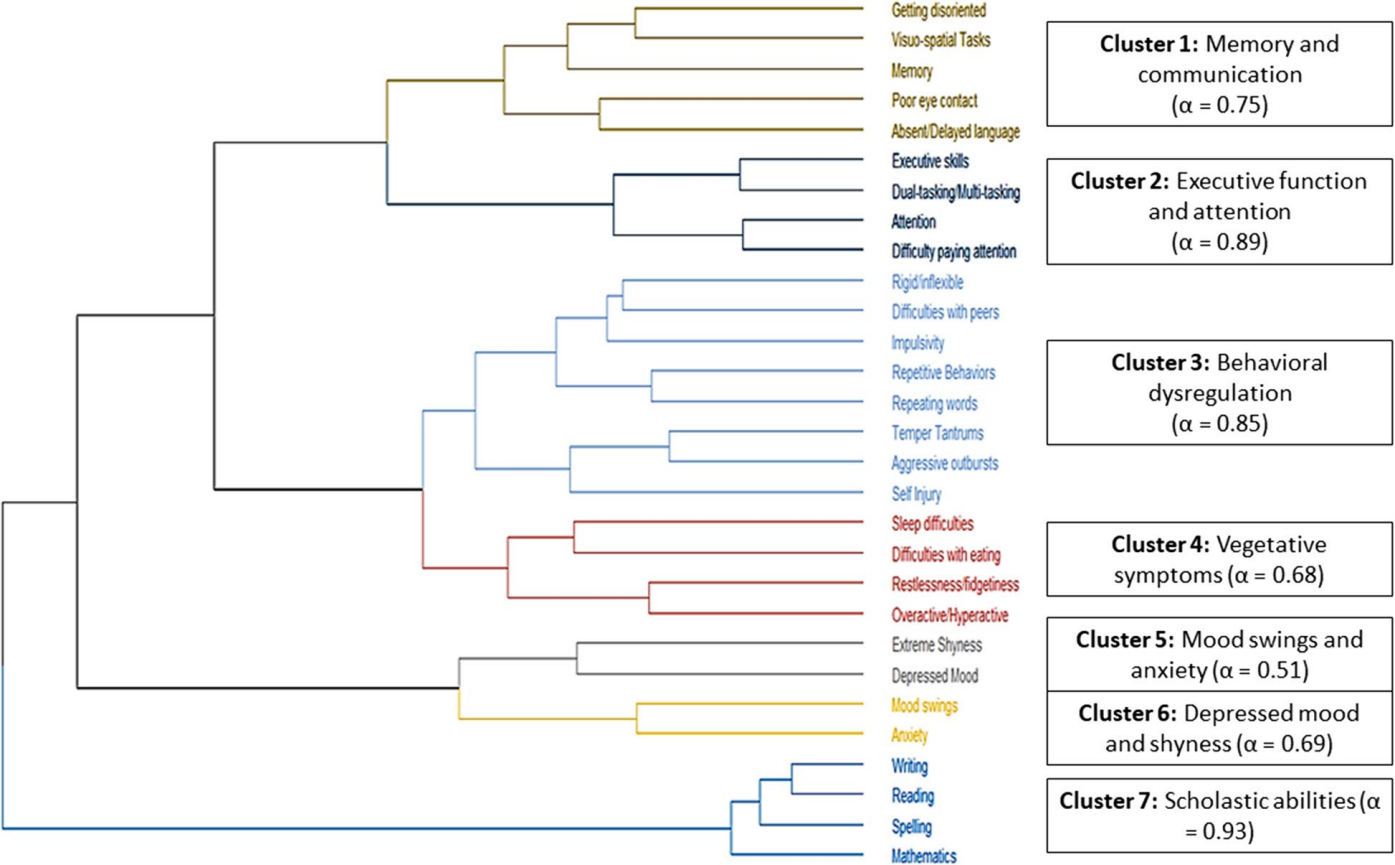
Cluster analysis (*Cronbach alpha is on the right*)

The cluster analysis showed a seven-cluster solution to be optimal, with Ward’s method producing the most suitable cluster (dendrogram can be seen in Fig. 1). Methods like other hierarchical ones (including average, complete linkage, McQuitty), DIANA (divisive analysis) were discarded as they tended to have smaller clusters containing only one variable. Clusters 1 and 2 consisted primarily of difficulties with neuropsychological skills. The first was more broadly defined by symptoms of difficulties with memory/disorientation, visuo-spatial tasks, and language/communication including poor eye contact and second was more narrowly defined by difficulties with executive skills, dual tasking/multitasking, and attention as well as attentional behavioral difficulties that demonstrated even greater internal consistency than Cluster 1 (Cronbach *α* of 0.75 for Cluster 1 vs. α of 0.89 for Cluster 2). Cluster 3 was defined by symptoms of behavior dysregulation that included temper-tantrums, aggressive outbursts, self-injury, rigidity, difficulties with others, impulsivity, repetitive behaviors, and repeating words/phrases, with similarly high internal consistency (α of 0.85). Cluster 4 featured vegetative symptoms, such as sleep and eating, as well hyperactive symptoms like overactivity and restlessness (*α* of 0.68). Clusters 5 and 6 were associated with mood symptoms, the first centered around depressed mood and shyness (*α* of 0.51), and the other around mood swings and anxiety (*α* of 0.69). The final cluster (Cluster 7) consisted of difficulties at the scholastic level, including reading, writing, spelling, and mathematics and demonstrated the highest internal consistency overall (*α* of 0.93).

### Factor analysis

A total of 268 combinations of factor extraction and rotations were examined. The solution most similar to our cluster analysis (the 7-cluster Ward analysis) using the Tucker index to assess congruity was a seven-factor principal axis analysis with Promax rotation and load threshold of >0.35 (Table 4). Three items cross-loaded onto multiple factors; namely, attention difficulties both as a behavior symptom (onto Factors 1 and 2) and as a neuropsychological symptom (onto Factors 2 and 5) and executive skills (onto Factors 1 and 2), all with loadings of >0.35. Three symptom variables did not strongly correlate with any one factor: difficulties with eating, difficulties with sleep, and difficulties with peers.

**Table 4:**
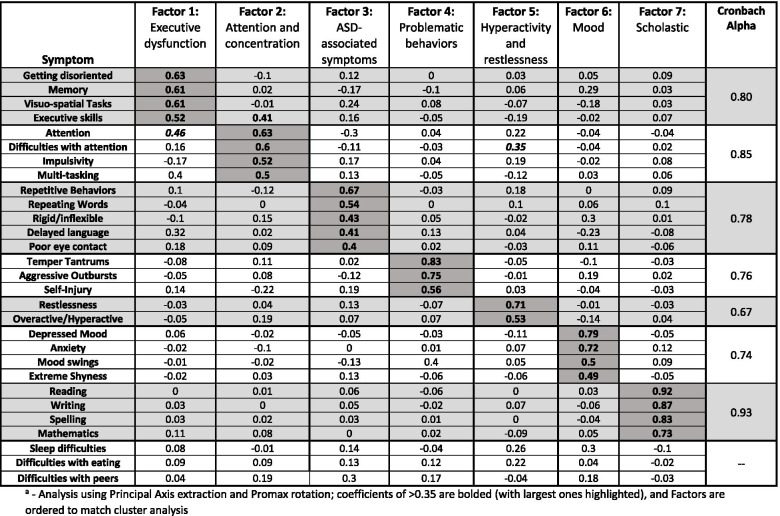
Factor analysis

Analysis using Principal Axis extraction and Promax rotation; coefficients of >0.35 are bolded (with largest ones highlighted), and factors are ordered to match cluster analysis

Overall, comparison of the factor analysis with the clustering analysis generally revealed similar groups of variables, confirming which specific factors were primary drivers for clustering group identity. For example, scholastic variables completely grouped together in both analyses (Cluster 7 and Factor 7) and, while mood symptoms loaded onto one single factor (Factor 6), they were the exact same symptoms that separated into the two closely related cluster groups for mood (Cluster 5 and Cluster 6). Neuropsychological skills of Factors 1 and 2 mapped primarily onto the clusters for memory and communication (Cluster 1) and executive function/attention (Cluster 2), respectively. Problematic behaviors (Factor 4) mapped exclusively to the cluster for dysregulated behaviors (Cluster 3), along with symptoms traditionally associated with autism spectrum disorder (ASD), such as repetitive behaviors, repeating words, rigidity/inflexibility (Factor 3), and impulsivity (Factor 2). Delayed language and poor eye contact (Factor 3) mapped to the memory and communication cluster (Cluster 1), and hyperactivity and restlessness symptoms (Factor 5) mapped to the vegetative symptoms cluster (Cluster 4).

### K-means cluster analysis

To extend our analysis of TAND symptom clusters and the factors driving each symptom cluster, we next wanted to identify natural phenotypic presentations in individual patients (i.e., which symptoms are most likely to co-present within the same individual that in combination define the overall TAND profile of that individual patient). To this end, each of the 26 symptom variables assigned to a specific factor (see Table 4) and each factor within each individual participant was given a factor score between 0 and 1 (number of symptoms present divided by the total number of symptom variables comprising that factor). Participants were excluded from this analysis if any of the variables were missing (*n*=26 of 668 participants, or 4%), as this would mean a factor score could not be calculated. A K-means seven-cluster solution was found. Factors with an average factor score >0.5 (meaning that individuals in that group were reporting more than 50% of symptoms) were considered a defining feature for that K-cluster profile. Attempts at reducing or increasing variance in factor scores created additional small splinter profile clusters or overlapping profile clusters with similar factor scores, respectively.

As shown in Table 5, TAND profiles with high symptom burden (five or more defining factors) were the most prevalent (*n*=249/642, 39%). Within this larger group with high symptom burden, three separate K-cluster profiles were identified, consisting of all factors (*n*=127), all factors except scholastic (*n*=58), or all factors except mood and problematic behaviors (*n*=64). TAND profiles with intermediate symptom burden (*n*=195/642, 30%) could be similarly divided into three separate K-clusters profiles, all of which were characterized by difficulties with inattention/concentration and one additional factor each (scholastic (*n*=63), mood (*n*=69), and executive function (*n*=63)). The final TAND profile was the low symptom burden group, with none of the seven defining factors reaching the >0.5 threshold (*n*=198/642, 31%).

**Table 5:**
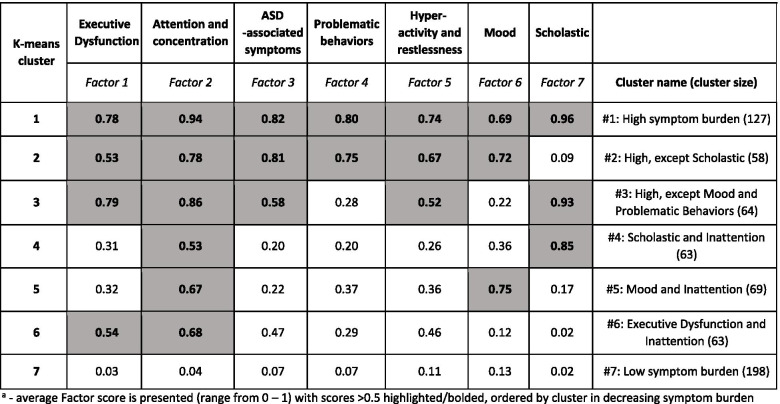
K-means cluster analysis

Average factor score is presented (range from 0 to 1) with scores >0.5 highlighted/bolded, ordered by cluster in decreasing symptom burden

We explored the influence of sex, age, and seizure frequency for each of the seven TAND profiles identified by the K-means cluster solution, as well as the parent/caregiver/self-reported impact score (0–10) of TAND (Table 6). Seizure frequency was analyzed by calculating monthly seizure frequency for each participant at time of completion of the TAND Checklist. Overall, there was statistical difference between groups by one-way ANOVA for each of these characteristics. Subsequent post hoc comparison of each profile with each other found notable differences, including significantly more males, seizure frequency, and parent/caregiver/self-reported impact scores in the high symptom profile groups compared to the low-profile group (*p < 0.05*). Within the TAND profile groups with intermediate symptom burden, the age was significantly higher in the intermediate profile group defined by mood difficulties and inattention/concentration when compared to the other profile groups (*p < 0.05*). Seizure frequency was lowest for TAND profile groups with intermediate symptom burden limited to attention/concentration combined with either scholastic or mood symptoms only (*p < 0.05*).

**Table 6:** Comparison of profiles by sex, age, impact, and seizure frequency

	Profile 1 (***N*** = 127)	Profile 2 (***N*** = 58)	Profile 3 (***N*** = 64)	Profile 4 (***N*** = 63)	Profile 5 (***N*** = 69)	Profile 6 (***N*** = 63)	Profile 7 (***N*** = 198)	Total^a^ (***N*** = 642)
**Sex** (percent male)^b^	62%	50%	64%	46%	42%	57%	44%	52% (Χ^2^ = 17.9, df 6, *p = 0.006*)
	(5,7)*	--	(7)*	--	(1)*	--	(1,3)*	
**Age** (average, SD)^b^	16.5 (±11.0)	21.4 (±15.5)	16.2 (±10.7)	17.5 (± 9.6)	23.8 (±15.4)	13.3 (±13.7)	14.1 (±16.5)	16.7 (± 14.2) (F_6,635_ = 5.93, *p < 0.0001*)
	(5)**	(6,7)*	(5)**	(5)*	(6,7)*** (1,3)** (4)*	(5)*** (2)*	(5)*** (2)*	
**Impact** (average, SD)^b^	7.1 (±2.4) *n = 125*	5.9 (±2.8)	5.6 (±2.2)	4.2 (±2.5) *n = 62*	5.3 (±2.6) *n = 66*	4.6 (±2.6)	2.47 (±2.9) *n = 195*	4.69 (± 3.2) (F_6,626_ = 44.81, *p < 0.0001*)
	(3,4,5,6,7)*** (2)**	(4,7)*** (1)** (6)*	(1,7)*** (4)** (6)*	(1,2,7)*** (3)** (5)*	(1,7)*** (4)*	(1,7)*** (2,3)*	(1,2,3,4,5,6)***	
**Monthly seizure frequency** (average, SD)^b^	47.3 (±133.4) *n = 122*	35.5 (±92.7) *n = 56*	37.4 (±95.0) *n = 63*	5.0 (±15.8) *n = 62*	11.3 (±32.4) *n = 66*	58.8 (±98.9)	25.8 (±81.4) *n = 195*	31.7 (± 93.12) (F_6,620_ = 3.08, *p* = 0.006)
	(4)** (5)*	--	(4)*	(1,6,7)** (3)*	(6)** (1)*	(4,5)**	(4)**	

*p < 0.05, **p < 0.01, ***p < 0.001 (FDR-adjusted)

^a^Chi-square test for sex, one-way ANOVA for age, impact, and monthly seizure Frequency

^b^Sample size specified if different from profile size

## Discussion

Using the TAND Checklist, we report on the first study to identify distinct clinical profiles within the TSC population via cluster and factor analysis followed by K-means cluster analysis. The majority of participants were administered the TAND checklist at yearly intervals, which is in line with current recommendations [3, 5]. The rate of TAND symptoms reported over the three-year period of the study remained overall stable. There were important differences in rates of symptoms when individuals were divided by age (children vs adult). Children tended to have more external behavioral symptoms, while adults tended have more internal mood symptoms. This pattern was consistent with the large, multinational TOSCA registry involving 2093 participants [6] and is consistent with age-based expectations in psychopathology [15], as well as other neurodevelopmental disorders like ADHD [16].

Of note, our results overall found similar rates of TAND symptoms as those of previous studies, with approximately 90% of individuals reporting at least one TAND symptom [3, 17]. Some individual TAND characteristics in our cohort were nearly identical compared to TOSCA with reported rates for intellectual disability (54%), ASD (21%), and ADHD (20%) [18]. However, we found that rates for most mood and behavioral symptoms in our cohort were consistently higher (22–57% vs. 7–21% in TOSCA). These differences could not be explained by age or sex, even when splitting the cohorts between children and adults. We suspect the differences are better explained by the methodology used for data collection. Whereas our study relied solely on the TAND Checklist for data collection, TOSCA employed a variety of methods, including patient self-report on the patient experience and quality of life, review of hospital discharge and clinic visit files and electronic medical records, patient questionnaires, and ad hoc clinical databases [19]. Furthermore, the TAND Checklist was administered to every patient seen in our TSC clinic over the reporting period while TAND-related data was reported missing for 25–40% of participants of TOSCA [18] despite the wide net for data collection used. To investigate these differences further, TAND Checklist responses from our cohort could be directly compared with the subset of TOSCA participants prospectively assessed using the TAND Checklist (*n*=85), results of which were published in 2020 but did not include individual checklist item frequency rates [9]. Perhaps a greater influence on observed differences of TAND symptoms in our cohort compared to TOSCA is the widespread discrepancies in TAND assessment and treatment generally [3, 20], which may be minimized at our center by the universal adoption of the TAND Checklist into our clinical practice as recommended by the 2012 International Consensus Group Recommendations for TSC [5] that creates heightened awareness of TAND in patients/parents/caregivers seen at CCHMC and our team of TSC clinicians. Furthermore, inclusion of a developmental pediatrician, neuropsychologists, and psychiatrists into our multidisciplinary team of TSC specialists removes barriers to formal assessment for TAND and consultation with TAND specialists.

Intellectual disability is known to be highly prevalent in TSC, with a reported rate of 45% in toddlers at 24 months in the TSC Autism Center of Excellence Research Network (TACERN) [21] to 55% in patients of all ages in the TOSCA study across all ages in TOSCA. In our study, the rate of parent/caregiver/self-reported intellectual disability was 62%. Although formal evaluations of IQ were only available for 35% of our sample, participants with reported intellectual disability (estimated IQ < 70) were concordant with formal evaluations. Intellectual disability correlated with higher rates in most TAND symptoms reported via the TAND Checklist including psychiatric mood disorders and ASD, consistent with previous studies [22, 23]. It is therefore even more important for providers to be especially careful when assessing TAND symptoms in those with intellectual disability. We also found an association between intellectual ability and ADHD diagnosis, though this was not found in other studies [23].

We identified seven TAND symptoms profiles occurring naturally in TSC, spanning the full spectrum of low to high symptom burden. The cluster and factor analysis used to identify these TAND symptom profiles were highly concordant in our study and similar to many aspects of the previous cluster analysis performed by Leclezio et al. in a pilot study of South African and Australian TSC patients (*n* = 56) [8] and a more recent analysis by de Vries et al. of TOSCA registry participants (*n*=85) [23]. All three studies found Ward’s method for hierarchical clustering best suited for identifying naturally occurring TAND symptom clusters in TSC. However, key differences are present. Our clustering solution identified 7 symptom groups, whereas the clustering solution of the two prior studies identified just 6 symptom groups. All three studies also identified scholastic, neuropsychological, and behavioral dysregulation symptom clusters, but individual TAND checklist items defining each symptom cluster frequently differed from study to study. This led to some unique findings in our analysis, such as the single neuropsychological cluster in the previous two studies being divided into separate memory/communication and executive function/attention clusters, and the single symptom cluster for mood/anxiety being divided into separate clusters for mood swings/anxiety and depressed mood/shyness. Perhaps the most surprising difference was the lack of specific ASD-like and hyperactive/impulsive clusters identified in both previous studies. Instead, these symptoms sorted in cohort within other clusters for the memory/communication, behavioral dysregulation, and vegetative symptoms. We cannot explain these differences other than the larger size of our cohort providing granularity not appreciated in smaller cohorts and the potential impact of differences that may be unique to our center to influence TAND symptom identification and reporting by patients, as discussed above. Valuable insight into these possibilities will be possible via analysis of TAND Checklist responses that are being collected in large, multicenter studies including the Developmental Synaptopathies Consortium of the Rare Diseases Clinical Research Network (clinicaltrials.gov NCT02461459) and the TANDem Consortium (tandemconsortium.org).

Poor sleep, difficulties with eating, and difficulties with peers did not load well on any individual factor driving individual cluster symptomology. In the studies by Leclezio et al *.*[8] and de Vries et al .[23], poor sleep loaded onto the factor best correlated with the mood/anxiety cluster and difficulties with eating and peers loaded onto the factor best correlated with the ASD-like cluster. Intuitively, sleep, eating, and interaction with peers may be closely connected with a variety of other symptoms investigated via the TAND checklist and therefore not closely loading with any one factor but rather many of them. Sleep disturbances in particular may be difficult to load on individual factors contributing to TAND symptomology as it may also be driven by factors fully independent of TAND. For example, circadian rhythms are known to be perturbed directly via the mTOR pathway [24], as well as influenced by epilepsy and anti-seizure medications [25].

We extended our study beyond the hierarchical cluster analysis and factor loading of individual symptoms to identify naturally occurring TAND patient profiles defined by the constellation of symptom clusters occurring together in individual patients. We found seven unique TAND patient profiles using this approach, ranging from high to low symptom burden. Approximately 20% of participants exhibited the highest symptom burden with involvement of all factors and another 19% exhibited high symptom burden involving five or more factors. These patients highlight the need for TSC specialists to be familiar with the full spectrum of symptoms contributing to each level of TAND (behavioral, intellectual and academic, psychiatric, neuropsychological and psychosocial), including their diagnosis and management. While 31% (*n*=198/642) of patients were classified as having the lowest symptom profile in which no single common factor was identified as responsible for their individual TAND profile, only one third of individuals in this group (*n*=66) had zero TAND symptoms reported at the initial visit. Therefore, individuals in this group still periodically warrant further evaluation as per current guidelines recommended [3, 5].

### Limitations and future directions

It is important to note that TAND Checklist responses from a cohort of 365 participants from CCHMC are included in a more recent validation study by *Leclezio* and colleagues [10] with some degree of participant overlap in the current study. Furthermore, our study exclusively focused on TAND Checklist responses and did not attempt to correlate TAND Checklist responses with other standardized neuropsychological or neurodevelopmental assessment tools. Finally, the TAND Checklist implementation utilized by our TSC Clinic at CCHMC is collected electronically via a tablet device completed by the parent/caregiver/patient before the clinician enters the exam room and reviews responses with the individual completing the checklist. This implementation facilitates increased adoption and TAND Checklist completion during the clinical encounter, but is non-standard in that it is not conducted via direct interview between the clinician and the parent/caregiver/patient as is recommended [3]. An updated version of the TAND Checklist-L used in the current study (TAND Checklist-SQ) is under development through the TANDem Consortium (tandemconsortium.org) with the aim to be completed by participants and their caregivers through a smart device application rather than solely through an interview, similar to how we utilize the current TAND Checklist at CCHMC with tablets. The TAND Checklist-SQ application will also have the added capability to capture both frequency and severity of TAND symptoms and provide useful information to users and clinicians for additional evaluation and treatment that is based on clinical consensus guidelines for the treatment of each TAND cluster [26].

Ongoing questions include investigating the dynamic nature of these phenotypic groups over time both at a group level and within individuals, determining if there are certain age and sex associations for these phenotypes, and how (if) these are associated with underlying TSC genotype and additional clinical disease manifestations of TSC. The ultimate goal is for a personalized TAND approach to individuals affected with TSC that can be implemented at an early age for prognostication and treatment and continued throughout the lifespan of individuals with TSC as additional components of TAND emerge and/or change.

## Conclusions

TAND represents a common and often burdensome aspect of TSC. Our findings validate previous research on natural clustering of TAND symptoms and identify distinct individual TAND symptom profiles defined by constellations of individual symptom clusters occurring together within similar individuals.

## Data Availability

The datasets during and/or analyzed during the current study available from the corresponding author on reasonable request.
